# Osseointegration of implants with superhydrophilic surfaces in rats with high serum levels of nicotine

**DOI:** 10.1590/0103-6440202305096

**Published:** 2023-05-15

**Authors:** Felipe Eduardo Pinotti, Maurício Andrés Tinajero Aroni, Guilherme José Pimentel Lopes de Oliveira, Bruno Luís Graciliano Silva, Elcio Marcantonio, Rosemary Adriana Chiérici Marcantonio

**Affiliations:** 1 São Paulo State University (Unesp), School of Dentistry, Araraquara, Brazil.; 2 San Francisco de Quito University (UFSQ), Quito- Ecuador.; 3 School of Dentistry at the Universidade Federal de Uberlândia, Brazil.

**Keywords:** implant surface, osseointegration, smoke

## Abstract

This study aimed to evaluate the effect of nicotine administration on the osseointegration of a superhydrophilic implants surface on rat tibiae. Thirty-two rats were used and divided into 2 groups according to the administration or not of nicotine: HH - Installation of implants with superhydrophilic surfaces in healthy animals; and HN - Installation of implants with superhydrophilic surfaces in animals subjected to nicotine administration. The animals were euthanized 15 and 45 days after implant placement (n = 8). Osseointegration was assessed by means of biomechanical analyses (removal torque), microcomputed tomography (volume of bone around the implants- %BV/TV), and histomorphometry (bone-implant contact -%BIC and the bone area between implant threads -%BBT). The animals subject to the nicotine administration presented lower removal torque than the control animals at the 45-day period (21.88 ± 2.80 Ncm vs. 17.88 ± 2.10 Ncm). The implants placed in the control rats presented higher %BIC (54.26 ± 6.59% vs. 39.25 ± 4.46%) and %BBT (50.57 ± 5.28% vs. 32.25 ± 5.24%) than the implants placed in nicotine animals at 15-day period. The nicotine administration reduces the osseointegration at 15 days, however, the superhydrophilic surface equalized the osseointegration in nicotine-exposed animals compared with healthy animals after 45 days of implant placement.

## Resumo

O objetivo deste estudo foi avaliar o efeito da administração de nicotina na osseointegração de implantes com superfície modificada por jateamento de oxidos e ataque ácido instalados em tíbia de ratos. Foram utilizados 32 ratos que foram divididos em 2 grupos de acordo com a administração ou não de nicotina: HH - Instalação de implantes com superfícies superhidrofílicas em animais sadios; e HN - Instalação de implantes com superfícies superhidrofílicas em animais submetidos à administração de nicotina. Os animais foram eutanasiados 15 e 45 dias após a colocação do implante (n = 8). A osseointegração foi avaliada por meio de análises biomecânicas (torque de remoção), microtomografia (volume de osso ao redor dos implantes - %BV/TV) e histomorfometrica (contato osso-implante -%BIC e área óssea entre roscas do implante -%BBT). Os animais submetidos à administração de nicotina apresentaram menor torque de remoção do que os animais controle no período de 45 dias (21,88 ± 2,80 Ncm vs. 17,88 ± 2,10 Ncm). Os implantes colocados nos ratos controle apresentaram maiores %BIC (54,26 ± 6,59% vs. 39,25 ± 4,46%) e %BBT (50,57 ± 5,28% vs. 32,25 ± 5,24%) do que os implantes colocados nos animais submetidos a administração de nicotina no período de 15 dias. A administração de nicotina prejudicou a osseointegração no período de 15 dias. Entretanto, a superfície superhidrofílica equalizou a osseointegração em animais expostos à nicotina em comparação com animais saudáveis após 45 dias da colocação do implante.

## Introduction

Oral rehabilitation with osseointegrated implants has achieved good outcomes in several clinical conditions regardless of the size of the rehabilitation ^1^. However, some host factors may directly interfere with host bone tissue metabolism, which can negatively affect the implant osseointegration process and impair the long-term maintenance of the implants^2^.

Smoking is one of the most important risk factors related to tooth loss^3^ ^,^ ^4^. Previous clinical studies have shown that smokers are approximately 2 to 3.6 times more likely to lose teeth^3^. Thus, this population would have a greater need to replace lost dental elements with osseointegrated implants. However, this same habit is also related to problems in achieving good osseointegration, as well as in relation to the host's resistance to the microbial challenge that can result in peri-implant diseases^4^. Indeed, previous clinical studies have been shown that the dental implants lost is 5.64 times higher in smokers^5^ while the occurrence of periimplantitis is 2.63 higher in smokers compared with non-smokers^6^. These deleterious effects have been related to the high number of cigarettes used per day and heavy smokers (>10 cigarettes per day) are in a higher risk for implants lost ^4^. Furthermore, these outcomes are related to the effects of smoking on the inhibition of macrophages, decreased chemotaxis of inflammatory cells, and decreased platelet aggregation, which impair the host's immune-inflammatory response^7^.

Among the numerous toxic substances present in cigarettes, nicotine stands out for being the substance related to addiction among its consumers^8^, as well as to important changes related to the healing of tissues(9, 10). Nicotine interferes with the activity of fibroblasts^10^ and osteoblasts^9^, generating less bone formation around the implants and decreasing their osseointegration^11^.

One approach used to accelerate the osseointegration process in individuals with challenging systemic conditions is the modification of the implant surfaces^12^ ^,^ ^13^. These physicochemical surface modifications aim to increase the stability of the blood clot that serves as a guide for the beginning of the osseointegration process, promoting an increase in their osteoconduction properties^14^ ^,^ ^15^. A surface modified by sandblasting and acid etching and maintained in sodium chloride solution has been shown to accelerate and improve the osseointegration process. These events have been related to the maintenance of the oxide layers promoted by the presence of the sodium chloride solution being in contact with the surface until the moment of implant installation, which maintains the oxide layer and increases the surface wettability^12^ ^,^ ^13^ ^,^ ^14^. In this context, this superhydrophilic surface may be an interesting alternative to improve the osseointegration in smokers.

Thus, the objective of this study was to evaluate the effect of the nicotine administration on the osseointegration of an implant surface modified by sandblasting, acid etching and maintained in an isotonic solution (superhydrophilic) in rat tibiae.

## Materials and methods

### Study design and ethical considerations

This study was approved by the Ethics Committee of Animal Use in Research (CEUA-44/2017). In this study, thirty-two male rats (*Rattus norvegicus*, albinus variation: Holtzman) of approximately 3 months of age, with a body mass between 200-300 grams, were kept in the Vivarium of the our institution. The animals were fed solid commercial rat chow and had access to water *ad libitum* before and throughout the experimental period in an environment with controlled water, light and temperature.

The 32 animals were randomly divided into 2 groups with 16 animals each, which were evaluated in two experimental periods (15 and 45 days), with 8 animals in each group. The groups were divided according whether the animals were subjected to challenge with nicotine: HH: Systematically healthy animal subjected to implant installation with a superhydrophilic surface created by sandblasting and acid etching and maintained in a sodium chloride solution (ACQUA Surface, Neodent®, Curitiba, PR, Brazil); HN: Animal that received nicotine administration and an implant with a superhydrophilic surface (ACQUA Surface, Neodent®, Curitiba, PR, Brazil).

Nicotine administration

Nicotine administrations were performed through daily subcutaneous applications every 12 hours in the dorsal region of the animals, starting 30 days before the surgical procedure. The animals received a diluted solution of nicotine of 5 mg/ml, which was administered a dose of 3 mg/kg^16^. The animals in the control group received administrations of saline solution at the same frequency as the animals subjected to the challenge with nicotine. The nicotine and saline solution administrations were maintained until the animals were euthanized.

### Surgical Procedure

The animals were anesthetized by a combination of ketamine (Agener União Ltda, São Paulo, SP, Brazil) and xylazine (Rompum, Bayer SA, São Paulo, SP, Brazil). The implants (4 mm height and 2.2 mm diameter titanium implant, Acqua Implants, Neodent®, Curitiba, PR, Brazil) were placed in the rat tibiae through the application of a progressive sequence of drillers (2.0 mm spiral drill- Neodent®; Curitiba, PR, Brazil) with the aid of an electric motor, adjusted to 1200 rpm, under abundant irrigation with sterile saline solution. The implant was installed with the help of a digital key (Hexagonal digital key 1.2 mm - Neodent, Curitiba, PR, Brazil). Then, the soft tissues were sutured. The animals received, in a single dose, penicillin associated with streptomycin (Multibiotic Small, Vitalfarma, São Sebastião do Paraíso, MG, Brazil) and ketoprofen (Ketoflex; Mundo Animal, São Paulo, Brazil) intramuscularly. The animals were euthanized by an anesthetic overdose after 15 and 45 days of the implant’s placement. The tibiae were randomly separated, one of which was used for microcomputed tomographic and histomorphometric analyses, while the other was used for biomechanical analysis.

### Biomechanical analysis

After euthanasia, the tibiae were stabilized in a small vice. A hexagonal wrench was connected to both the implant and a torque wrench (Tohnichi, model ATG24CN-S, Tokyo, Japan - with a graduated scale of 0.05 N/cm, measuring the strength from 3 to 24 Ncm), and anti-clockwise movement was performed with the objective of unscrewing the implant. The maximum peak required to move the implant was noted as the removal torque value (Ncm).

### Microtomographic analysis

**Figure 1:**
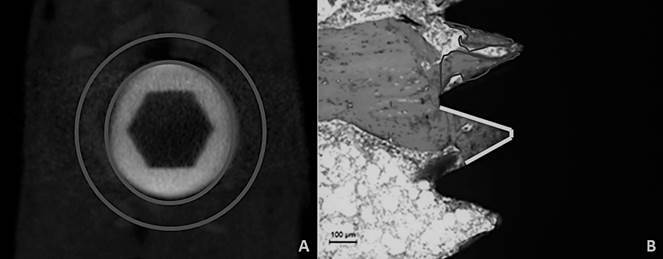
Scheme of evaluation of the micro-CT and histomorphometric analysis. A) The region of interest (ROI) was defined as a circular region 0.5 mm around the entire diameter of the implant (Area between the two circles); B) The percentages of bone-implant contact (% BIC) (Delimited lines) and the bone area between turns (% BBT) (area inside the region) were evaluated separately in the first three threads of the implants.

Tibiae that did not undergo biomechanical analysis were fixed in 4% paraformaldehyde for 48 hours and later stored in 70º alcohol. The samples were scanned by a microcomputed tomograph (Skyscan, Aatselaar, Belgium) with the following parameters: camera pixel: 12.45; X-ray tube power: 65 kVP, X-ray intensity: 385 µA, integration time: 300 ms, filter: Al-1 mm and voxel size: 18 µm^3^. The images were reconstructed, spatially repositioned and analysed by specific software (NRecon, Data Viewer, CTAnalyser, Aatselaar, Belgium). The region of interest (ROI) was defined as a circular region 0.5 mm around the entire diameter of the implant. This ROI was defined as the total volume (0.5 mm margin around the implants - 4.5 mm x 3.2 mm) (Figure 1A). The threshold used in the analysis was 25-90 shades of grey, and the values of the volume of mineralized tissue around the implants were obtained as a percentage (BV/TV%)^12^. A trained examiner who was blinded to the experimental groups performed this FEP analysis.

### Histomorphometric analysis

After scanning, the tibiae were dehydrated in a graded series of ethanol (60 - 100º), infiltrated, and polymerized in light-cured resin (Technovit 7200 VLC, Kultzer Heraus GmbH & CO, Wehrheim, Germany). The blocks containing the implant and bone tissue were cut at a central point using a cut and wear system (Exakt Apparatebeau, Hamburg, Germany). The sections were approximately 45 μm thick, stained with Stevenel blue associated with acid fuchsin and analysed under an optical microscope (DIASTAR - Leica Reichert & Jung products, Wetzlar, Germany) at 100X magnification. Histomorphometric evaluations were performed with ImageJ software (San Rafael, CA, USA). The percentages of bone-implant contact (% BIC) and the bone area between threads (% BBT) were evaluated separately in the first three threads of the implants (Figure 1B). A blinded and trained examiner (FEP) performed these analyses.

### Sample size calculation

The sample size calculation was performed take in consideration that the %BIC is the primary variable of this study. The differences between the groups considered as relevant was 10%. Then, considering that the standard deviation expected for this analysis is 4.63 ^12^ and establishing the type I error at 0.05 and β power at 0.90, indicates that the minimum number of animals in each group is 8.

### Statistical analysis

The distribution of the data generated in this study was assessed using the Shapiro-Wilk normality test. All data in this study were distributed according to normality; thus, parametric tests were applied for inferential analysis of the data. Comparison between the groups and between different periods within each group was performed using the unpaired t-test. GraphPad Prism 6 software (San Diego, CA, USA) was used to perform the statistical analysis, and all statistical tests were applied at a significance level of 5%.

## Results

### Biomechanical analysis

**Figure 2:**
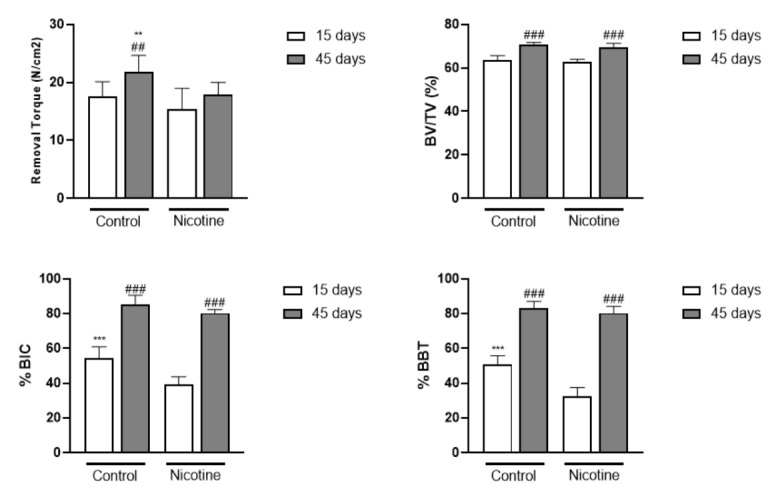
Representative graphs showed the mean and standard deviation of all the analysis performed in this study. **p<0.01; ***p<0.001- Differences between the control and nicotine animals; ##p<0.01; ###p<0.001- Intragroup differences regarding the experimental period - Unpaired t-test.

There was a progressive increase in the removal torque of the implants in the control animals over the 45-day period compared to the 15-day period (p <0.01). The animals subject to the nicotine administration presented lower removal torque than the control animals at the 45-day period (21.88 ± 2.80 Ncm vs. 17.88 ± 2.10 Ncm) (Figure 2).

### Microcomputed tomographic analysis

There was a progressive increase in BV/TV% in all groups over the 45-day period compared to the 15-day period (p <0.001). However, there were no differences in BV/TV% between the control and nicotine animals (Figure 2).

### Histomorphometric analysis

**Figure 3:**
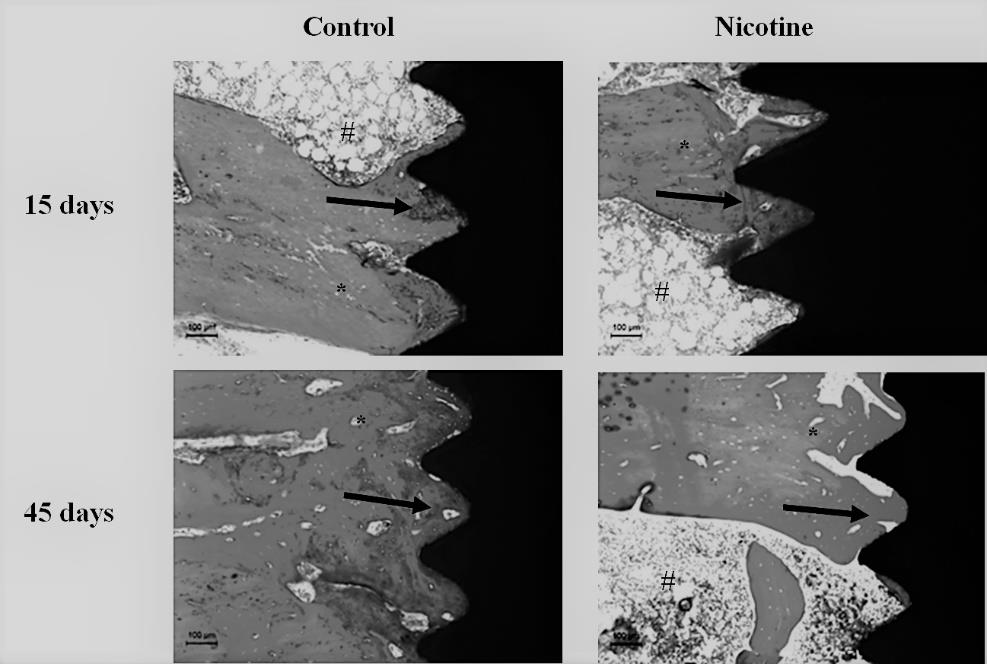
Representative images of the non-decalcified histological sections in all groups and experimental periods. It was possible to observe a higher degree of osseointegration in control animals at the 15-day period (Black Arrows). The amount of bone in the vicinity of the implants was also more noted at the surfaces in control animals (*). On the other hand, more soft tissues were observed in nicotine groups (#).

There was a progressive increase in the %BIC and %BBT values in all groups at the 45-day period compared to the 15-day period (p <0.001). In general, the implants placed in the control rats presented higher %BIC (54.26 ± 6.59% vs. 39.25 ± 4.46%) and %BBT (50.57 ± 5.28% vs. 32.258 ± 5.24%) than the implants placed in nicotine animals at 15-day period (Figure 2). Figure 3 shows representative images of the non-decalcified histological sections in all groups and experimental periods.

## Discussion

The results of this study were encouraging regarding the use of implants with superhydrophilic surfaces due to the benefit of the osseointegration process since the %BIC and %BBT were similar in longer experimental periods (45-days). However, the osseointegration occurred slower in nicotine animals and this fact may influence on the loading protocols of dental implants.

The analysis of the removal torque, %BIC, and %BBT demonstrated that the animals subjected to nicotine application presented less implant locking, less bone-implant contact, and less bone between the threads of the implants. These findings corroborate previous results presented in the literature that demonstrated that nicotine and smoking impair the bone healing process in bone fractures^17^, in skin, and in mucous membrane wounds^7^ due to a series of changes, both local and systemic in the body, including macrophage inhibition, decreased inflammatory cell chemotaxis, and decreased platelet aggregation, which impair the host's immune-inflammatory response^4^ .

Specifically, in bone tissue, nicotine has been shown to reduce osteogenesis by inhibiting the expression of BMP2 and VEGF^9^ and increasing vasoconstriction^4^, in addition to impairing the quality of blood clot formation^7^. Previous studies have shown how the aforementioned effects negatively interfere with bone repair in different experimental models. A preclinical study showed that nicotine administration to rats harmed bone repair in areas grafted with autogenous bone^16^. A systematic review of preclinical studies has shown that animals subjected to nicotine administration presented a reduced bone-implant contact of implants compared to healthy animals^11^. On the other hand, a preclinical study demonstrated that there was no deleterious effect of nicotine on the osseointegration process. However, the administration of this medication began just after implant placement, and this study design limits the applicability of these findings since patients will not suddenly initiate smoking habits after the surgical procedure if they do not have this habit before the intervention^18^.

Another important finding in this study was the reversed of the harmful effects of nicotine, especially at the longest experimental period (45 days), provided by the superhydrophilic surfaces. This good outcome of superhydrophilic surfaces is related to their high degree of wettability, which increases adhesion, differentiation, and osteoblastic activity^19^. Indeed, preclinical studies have shown that superhydrophilic surfaces improve osseointegration in native^13^ and grafted bone^12^. Superhydrophilic surfaces improved the osseointegration of implants placed in rabbit tibiae 28 days after the surgical procedure compared with a control surface^15^, improved the osseointegration of the tibiae of rats grafted with osteoconductive bone substitutes 15 and 45 days after implant placement^12^, and improved the osseointegration 90 days after implant placement in diabetic pig calvaria compared with control surfaces^20^. In healthy humans, an improvement in the osseointegration process has also been demonstrated in implants with superhydrophilic surfaces compared to implants with control surfaces after 2-4 weeks of implant placement^14^.

Despite the improvement in the %BIC and %BBT at the 45-days periods, the removal torque was lower in nicotine rats in this same period. Since the bone formation occurred in slow rats in the nicotine animals^21^, it is possible that the bone around the implants observed at the 45-days was more immature in nicotine than in control animals and this may be the reason for the lower biomechanical entanglement observed in the nicotine animals. All these findings suggest that the immediate and early load in dental implants with superhydrophilic surfaces placed in smokers must be avoid.

An important fact is that the systemic changes induced by nicotine can influence the host immunoinflammatory response for a long-term and the implants placed in smoking patients have lower success and survival rates than in non-smoking patients^6^. This host response alteration observed in smokers increases their susceptibility to presents a more prevalent and severe periodontitis and periimplantitis^4^. Although this aspect has not been tested in this study, it is possible that the superhydrophilic surface will increase resistance to the establishment and progression of peri-implant disease. This hypothesis must be tested in future studies.

The quest to improve the osseointegration process in smoking patients can not only start with interventions to improve the surfaces of the implants but also with approaches to induce the patient to quit smoking to benefit the osseointegration process^6^. A preclinical study showed that when rats that inhaled cigarette smoke received implants in their tibia, the cessation of this inhalation during the healing period was sufficient to improve the osseointegration process^22^. Other clinical studies have shown that quitting smoking benefits the treatment of periodontal disease and improves periodontal parameters in these populations^23^. However, the effect of quitting smoking on osseointegration in humans lacks further information and confirmation.

This study has as a limitation in that only nicotine was used to disrupt the osseointegration process to mimic the condition of active smokers. However, several other toxic substances can be formed during smoking, and the effect of all of these associated substances can be even more harmful to the osseointegration process that was demonstrated in this study. Other experimental models that mimic the smoking habit simulate the inhalation of smoke^24^, which also has limitations in mimicking a passive smoker condition Despite these limitations, the model used mimics the systemic effects caused by smoking in the osseointegration process, as demonstrated in a previous systematic literature review^11^. Furthermore, the parameters tested in this pre-clinical study provides limited information about the clinical outcomes regarding the osseointegration of dental implants, and the hypothesis that the superhydrophilic implants surfaces improve the osseointegration in smokers must the tests in future clinical studies.

## Conclusion

The nicotine administration reduces the osseointegration at 15 days. The superhydrophilic surface equalized the osseointegration in nicotine-exposed animals compared with healthy animals after 45 days of implant placement.
